# Complex Odontomas Hampering Eruption of Permanent Tooth

**Published:** 2014-07-20

**Authors:** Abdulla Mufeed, Abdul Hafiz, Ahammed Noufal

**Affiliations:** 1 Department of Oral Medicine; Radiology, Dental College, Perinthalmanna Malappuram Kerala, India; 2 Department of Pediatric Dentistry; 3 Department of Oral Pathology, Dental College, Perinthlamanna, India

**Keywords:** Odontomas, Tooth Eruption, Incisor, Odontogenic Tumors

Eruption of deciduous teeth, their exfoliation followed by eruption of permanent dentition is an orderly sequential and age specific event^[^^1^^]^. Significant deviations from accepted norms of eruption time are often observed. Most parents are anxious about delayed tooth eruption, as it is considered to be an important milestone during child’s development. Delayed tooth eruption might be the primary or sole manifestation of local or systemic pathology^[^^2^^]^. The systemic conditions like malnut-rition, rickets, endocrinopathies, chemotherapy, cerebral palsy and low birth weight can lead to delayed tooth eruption. 

 Local factors delaying eruption may include presence of mucosal barriers, supernumerary teeth, odontogenic and non odontogenic tumors, local infections, injury and ankylosis of deciduous teeth. 

**Fig. 1:**
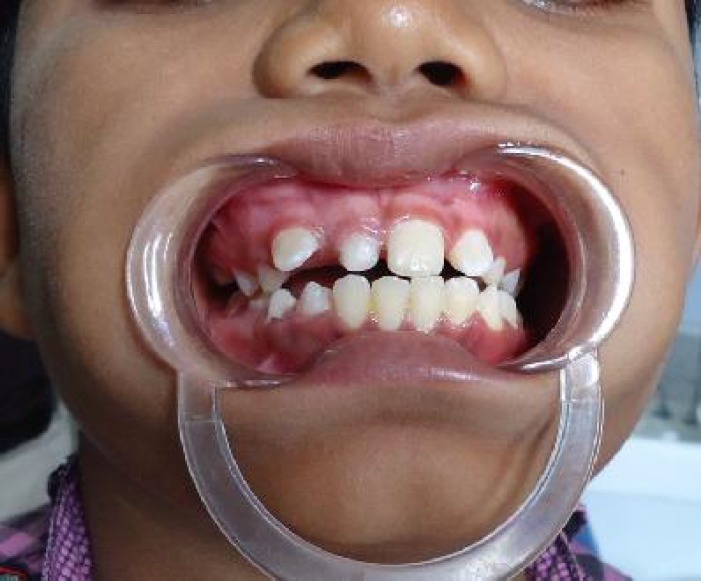
Intra oral view showing unerupted permanent incisor and drifting of adjacent permanent lateral incisor

**Fig. 2:**
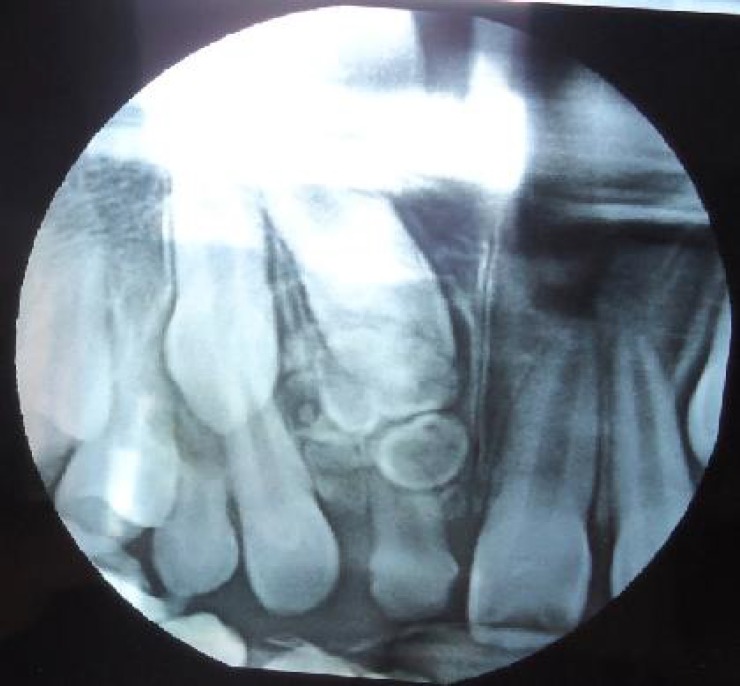
Radiograph showing presence of multiple radiopaque masses and retained permanent incisor

 A 12 year old boy referred with a complaint of missing right central incisor tooth. Examination of his upper jaw revealed that the central incisor tooth was unerupted and the deciduous counterpart was retained with mesial drifting of the lateral tooth creating an anesthetic appearance (Fig. 1). Radiographic examination showed presence of multiple circular and irregularly shaped radiopaque structures in anterior maxilla, overlapping the crown of unerupted permanent tooth (Fig. 2). Based on the diagnosis of complex odontoma, the area was surgically explored and the tumorous masses were removed. The gross examination and histological evaluation confirmed the diagnosis of complex odontoma. Patient is presently under observation as parents opted to wait and orthodontic extrusion is planned if no physiologic eruption took place in 6 months post operative period. Odontomas are *mixed odontogenic tumors*, since they are composed of both epithelial and mesenchymal tissues. However, biologically regarded as hamartomas rather than neoplasms^[^^3^^]^. Most of the cases of odontomas are often undetected because they are clinically asymptomatic and nonaggressive. They are usually identified on routine radiographic examinations or during evaluation of delayed tooth eruption as in this case.

 A thorough visual, manual and radiographic examination should be performed for all pediatric patients who present with clinical evidence of delayed eruption, missing or displaced tooth. 

Early identification and removal of odontomas help us to:

- Adopt less complex and invasive treatment
- Ensure better prognosis
- Avoid displacement or devitalization of adjacent tooth

After removal of the obstacle from the path of eruption, an impacted tooth either erupts spontaneously if it has conserved its eruptive force or orthodontic force is required to bring the tooth in normal position^[^^4^^]^. 
